# LCMS-Net: Deep Learning for Raw High Resolution Mass Spectrometry Data Applied to Forensic Cause-of-Death Screening

**DOI:** 10.1021/acs.analchem.5c05404

**Published:** 2026-02-27

**Authors:** Lisa M. Menacher, Liam J. Ward, Fredrik Heintz, Henrik Green, Oleg Sysoev

**Affiliations:** † Department of Computer and Information Science, 4566 Linköping University, 581 83 Linköping, Sweden; ‡ Department of Biomedical and Clinical Sciences, Linköping University, 581 83 Linköping, Sweden; § Department of Forensic Genetics and Forensic Toxicology, National Board of Forensic Medicine, 587 58 Linköping, Sweden; ∥ AI4x Center of Excellence, Linköping University, 581 83 Linköping, Sweden; ⊥ Department of Biomedical and Clinical Sciences, Science for Life Laboratory, Linköping University, 581 83 Linköping, Sweden

## Abstract

Current preprocessing workflows for untargeted metabolomics using liquid chromatography-high resolution mass spectrometry (LC-HRMS) are time-consuming and require significant domain knowledge. Furthermore, they lack reproducibility or may fail to detect some metabolites entirely. We introduce LCMS-Net, an end-to-end deep learning model for the analysis of LC-HRMS data, to address these challenges. LCMS-Net mitigates the need for manual data preprocessing by operating directly on the raw LC-HRMS data and explicitly modeling its spatial properties. The effectiveness of this fully automated workflow is shown through two case-studies, cause-of-death (CoD) screening and colon cancer detection. For the cause-of-death screening task, LCMS-Net achieved a 9% improvement in F1-score compared to the previous state-of-the-art model (OPLS-DA). For the colon cancer detection task, LCMS-Net achieved an F1-score improvement of 1.8% compared to the previous state-of-the-art model (DeepMSProfiler). Furthermore, LCMS-Net significantly reduces batch effects that are a common source of bias in metabolomics data analyses. This was shown by using a training and test set from different measurement instruments, where the performance only differed by at most 3% as to using data from the same instrument. Compared to other end-to-end deep learning methods for LC-HRMS data, LCMS-Net is also structurally simpler and does not rely on pretraining, which makes it faster and computationally more efficient.

## Graphical abstract

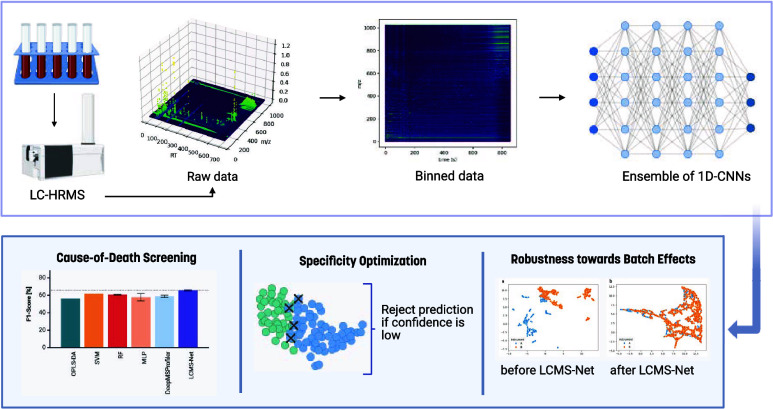

## Introduction

Metabolomics is the study of all low-molecular-weight substances in a biological specimen.^1^ As this is closely linked to cellular functions and biochemical processes, it offers a comprehensive view of the current physiological state of an organism. This makes metabolomics a promising tool for a broad range of applications, including diagnostics, precision medicine, and toxicology.^1–3^ However, large-scale large-scale liquid chromatography-high resolution mass spectrometry (LC-HRMS) studies are difficult due to the complex and labor-intensive data preprocessing. This typically involves peak picking, peak alignment, gap filling, and normalization, to simplify the data structure, extract biologically relevant features, and improve the interpretability for subsequent analyses.^4–7^ Although tools like XCMS or MZmine offer widely used implementations of this workflow, the preprocessing of LC-HRMS data remains challenging, for a number of reasons: (1) The preprocessing of LC-HRMS data can be time-consuming due to computational constraints for large datasets.^4,8,9^ (2) Domain knowledge is necessary to select suitable parameters for the used preprocessing software, and thus to obtain reliable features.^4,7,8^ (3) The reproducibility of the extracted features is often limited across software libraries, especially for low-abundant metabolites.^4,8^ (4) Preprocessing tools may fail to detect some metabolites entirely, resulting in the loss of potentially important information.^4^

In the past few years, deep learning models have emerged as promising alternatives to the traditional preprocessing workflow for LC-HRMS data. While many publications in this field have been focused on enhancing a particular step of the preprocessing, such as peak identification or peak alignment, end-to-end deep learning has also gained popularity.^9,10^ In end-to-end deep learning, a model is trained to extract useful information directly from raw data without explicit feature engineering. Often, this is less time-consuming than conventional methods and improves the predictive accuracy of the downstream task. This was demonstrated by Cadow et al., who converted raw MS data from prostate biopsies into pseudoimages through binning. They then applied pretrained deep learning models for image processing to extract feature vectors from the pseudoimages and trained a classifier to discriminate between tumor and healthy cases.^11^ A similar approach was also used by Shen et al. to predict the gestational age of pregnant women. To address data scarcity, they proposed a data augmentation strategy that simulates retention time (RT) drifts during the data acquisition. As a result, several pseudoimages can be produced from a single LC-HRMS sample, which substantially increases the size of the training set.^12^ Furthermore, Wang et al. developed an end-to-end deep learning model to detect esophageal squamous cell carcinoma based on raw LC-HRMS data. By decomposing a sample into multiple tiles instead of just one down-scaled pseudoimage, their approach preserves more details of the raw LC-HRMS data.^13^ Most recently, Deng et al. used an ensemble of pretrained image classification models for lung cancer detection. Their approach enables more robust predictions for multiclass classification tasks. Furthermore, model explainability was addressed through the analysis of contributing factors.^14^ Although, these studies collectively highlight the potential of end-to-end deep learning for metabolomics-based analyses, they also show its limitations. For example, the use of pretrained architectures for image classification may be flawed due to the substantially different topology of LC-HRMS data and natural images. Natural images typically contain hierarchically structured objects with geometrical properties such as size or patterns while objects in LC-HRMS pseudoimages are essentially represented as one-dimensional peaks along the retention time axis and are sharply localized in *m*/*z*-value.^6,15^ Existing deep learning methods for metabolomics primarily use 2D-convolutions and thus do not take into account such spatial properties of LC-HRMS data. Consequently, there remains a need for more robust methods that are specifically tailored to LC-HRMS data. This is particularly relevant for real-world applications, where batch effects and diverse study populations are common and difficult to mitigate.

One such application is post-mortem metabolomics, where samples collected after death are analyzed. Due to the nature of the subject, post-mortem samples are often highly heterogeneous in terms of age, sex, body mass index (BMI), previous diseases, and post-mortem interval (PMI). Moreover, they are typically collected over multiple years to obtain sufficiently large study populations, which introduces significant technical variation. At the same time, post-mortem metabolomics present a promising tool for death investigations.^3,16,17^ For example, several studies have investigated the use of post-mortem metabolomics for PMI estimation.^16,18,19^ Furthermore, several groups have explored the potential of post-mortem metabolomics for establishing cause-of-death (CoD) diagnoses and associated biomarkers.^3,20–25^ Among them, our group has shown that an OPLS-DA model trained with routinely collected femoral blood samples from autopsies can be used for high-throughput CoD screening.^26^ This is particularly relevant given the global decline in autopsy rates over the last decades.^27,28^ For instance, in 1999 in Sweden, clinical and forensic autopsies were performed for approximately 12% of all registered deaths. By 2018 this number had already declined to less than 6% of all registered deaths.^29^ With the potential use of post-mortem metabolomics for CoD screening, forensic pathologists could allocate their resources more efficiently by prioritizing the most critical cases and those with a high likelihood of foul play, while still maintaining broad coverage of unnatural deaths. Furthermore, accurate CoD diagnoses are essential for law enforcement, public health monitoring, and to support the grieving process of the deceased’s relatives.^29^ Thus, metabolomics-based CoD screening is a promising tool for providing additional insights to forensic pathologists and to increase the throughput of post-mortem examinations. However, further developments are needed to address its inherent challenges.

This study presents LCMS-Net, a fully automated end-to-end deep learning model for the analysis of LC-HRMS data. By directly processing the raw data and taking into account its spatial properties by using 1D-convolutions, LCMS-Net mitigates the need for time-intensive, manual feature engineering and improves the classification performance of downstream tasks. This is demonstrated through comprehensive benchmarking for two different applications, forensic CoD screening and colon cancer detection. To the best of our knowledge, it is the first time end-to-end deep learning is applied to post-mortem metabolomics and in the context of forensic science. Furthermore, LCMS-Net achieves robust results even in the presence of severe batch effects, as shown by training and evaluating the CoD screening model with data from different measurement instruments. Lastly, LCMS-Net does not rely on large pretrained deep learning models, and thus requires less computational resources than previous end-to-end learning approaches for the analysis of LC-HRMS data.

## Methods

### Sample Collection and Data Acquisition

Centroided LC-HRMS data collected from two experiments was used for the implementation and evaluation of LCMS-Net. The first experiment contains femoral blood samples from human autopsies, and the second experiment contains colon tissues samples from healthy and colorectal cancer (CRC) patients. The centroided LC-HRMS data was converted to open-source formats for easier processing in the subsequent steps. The CoD screening dataset were exported to mzData-files using MassHunter and the colon cancer dataset to mzML-files using ProteoWizard.^30,31^ Furthermore, the datasets were divided into a training and test set using a 75/25 split. To preserve group proportions across the subsets, stratified sampling was employed.

#### Cause-of-Death Screening Dataset

The cause-of-death screening study was approved by the Swedish Ethical Review Authority (Dnr 2019–04530 & Dnr 2025–0249–02). All samples were collected from autopsy cases admitted to the Swedish National Board of Forensic Medicine between July 2017 and December 2024, which had undergone routine toxicological screening via HRMS. Screening was performed from femoral whole blood samples, in accordance with standardized procedures reported previously.^32^ Sample separation was performed using gradient elution on a C18 column (150 mm × 2.1 mm, 1.8 μm; Waters Acquity HSS T3 column, Water Sverige AB, Sweden). MS-data was collected in positive mode and the total acquisition time for each sample was 12 min. Samples admitted between July 2017 and November 2020 were run using an Agilent 6540 Q-TOF system in MS-mode only, and samples admitted afterward using an Agilent 6546 Q-TOF system with data-dependent acquisition mode (autoMSMS). Aside from these differences in acquisition strategy, source settings and TOF parameters (e.g., gas temperatures, voltages, and mass ranges) were kept consistent between platforms. Henceforth, we will refer to the former as *Dataset A* and the latter as *Dataset B* for clarity.

Dataset A was primarily used for the implementation and evaluation of LCMS-Net and includes 4282 autopsy cases processed in over 641 analytical runs. All cases belong to one of the following five CoD groups: acidosis (*n* = 100), drug intoxication (*n* = 1385), ischemic heart disease (IHD) (*n* = 1362), hanging (*n* = 1200), and pneumonia (*n* = 235). A detail summary of the inclusion criteria and study population was previously described by Ward et al.^26^ Dataset B was used to evaluate the sensitivity of LCMS-Net to batch effects and includes additional 5485 autopsy cases with the same inclusion criteria and CoD groups as Dataset A.

#### Colon Cancer Dataset

For the cancer diagnosis task, a publicly available CRC dataset from MetaboLights (ID: MTBLS1129) was used. The dataset consists of 236 human colon tissue samples, of which 197 were diagnosed as tumorous and 39 as healthy. All samples were obtained from surgeries at the Memorial Sloan-Kettering Cancer Center (New York, United States) between 1991 and 2000. The sample collection and data acquisition was previously described.^33^

### Overview of LCMS-Net

LCMS-Net is a end-to-end deep learning model for the analysis of raw LC-HRMS data. First, a data preparation pipeline transforms the raw LC-HRMS data into pseudoimages. Afterward, convolutional neural networks (CNNs) specifically designed to take the spatial properties of LC-HRMS data into account are used to extract relevant features for the downstream task. Figure 1 shows an overview of this workflow for CoD screening, as well as the architecture of LCMS-Net.

**Figure 1:**
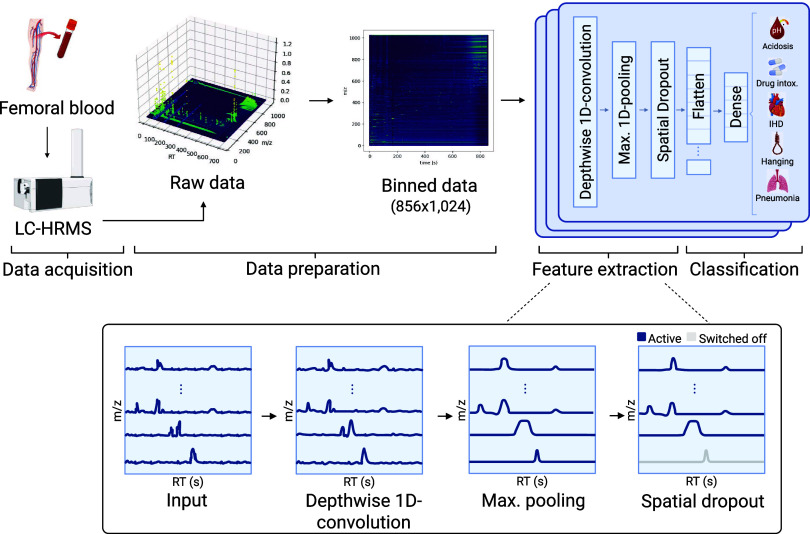
Overview of LCMS-Net applied to CoD screening. Binning is used to reduce the size of the raw LC-HRMS data and to ensure equally shaped input matrices. Afterward, the input matrices are processed by an ensemble of 1D-CNNs consisting of a depthwise 1D convolutional layer, followed by max pooling and spatial dropout. The results of the CNNs are flattened and dense layers are used to predict the class labels of the input data.

The data preparation pipeline is used to transform the raw LC-HRMS data into a structured and computationally feasible format that is suitable for deep learning. This is necessary, due to the large volume of the raw data that typically consists of thousands of data points, each defined by a RT, mass-to-charge ratio (*m*/*z*), and relative intensity. Previous studies have used data binning to achieve a more compressed and organized representation of the raw LC-HRMS data.^12–14^ Data binning maps each point of a sample into a predefined grid based on its RT and *m*/*z*, and then aggregates them by taking the maximum intensity value among all points within a bin. We refined this process for LCMS-Net by applying prior knowledge about the used LC-HRMS system and sample composition to adjust the resolution of the binning grid. Specifically, the width of a *m*/*z*-bin is defined so that regions with a high expected number of metabolites are divided into finer-grained bins, while regions with fewer expected metabolites are split into broader bins. Furthermore, the width of the RT-bins was set so that it mimics the sampling interval of the used measurement instrument, ensuring that each bin corresponds to the acquisition frequency of the instrument. This allows to capture more details than standard binning, while significantly reducing the size of the raw data. The required information for adaptive binning can usually be obtained through exploratory data analysis. However, if no prior knowledge is available, LCMS-Net can also be used with standard binning. Additional details of the binning process can be found in Supporting Text 1. Furthermore, Supporting Table 2 compares the prediction performance of LCMS-Net for adaptive and standard binning. After binning the raw LC-HRMS data, min-max scaling along the *m*/*z*-axis is applied to normalize intensity values, ensuring a consistent value-range across all samples.

Next, LCMS-Net employs an ensemble of 1D-CNNs to enhance generalization by integrating diverse feature representations learned by the individual neural networks.^34^ Predictions are obtained by averaging the outputted class probabilities from each ensemble member. First, the ensemble members apply depthwise convolutions across the RT-axis of the binned LC-HRMS data to extract local patterns. This also smooths the inputs and thus helps to mitigate the effects of RT shifts between samples. This is particularly important when the training dataset was collected over extended periods, during which physical changes of the measurement instrument or chemical changes of the chromatographic column might have occurred.^35^ Next, maximum pooling is applied to mimic the extraction of the strongest signals (i.e., peaks) for each *m*/*z*-bin, and spatial dropout to force the deep learning model to learn robust features instead of memorizing noninformative *m*/*z*-slices that contain primarily background noise. Lastly, the outputs of the feature extraction module are flattened and passed through a dense layer to predict the corresponding class label. To mitigate overfitting and improve convergence, batch normalization and L1-regularization are used. Furthermore, random oversampling and data augmentation are applied during the training of LCMS-Net to increase the robustness and reliability of its predictions. The latter simulates realistic variations in the LC-HRMS data by introducing random RT shifts of up to 10 s.^13^ A detailed evaluation of the effect of the different model components can be found in Supporting Text 2 and Table 2. Bayesian optimization was used to select the hyperparameters of LCMS-Net (see Supporting Table 3).

All code of LCMS-Net was implemented in the programming language Python (version 3.12.7). The open-source library pyOpenMS (version 3.2.0) was used to import the raw LC-HRMS data and TensorFlow (version 2.18.1) to create the deep learning model.

### Evaluation

#### Benchmark Models

Several machine learning models were selected as benchmarks to compare the prediction performance of LCMS-Net with the traditional workflow for the analysis of LC-HRMS data. For this purpose, a peak list was extracted from the raw LC-HRMS data using the R (version 4.1.2) library XCMS.^6^ The used parameters for the feature extraction can be found in Supporting Code 1. To handle missing values, half-minimum imputation was applied.^36^ Furthermore, the relative peak intensities were normalized using probabilistic quotient normalization (PQN), log-transformed, and scaled to unit variance.^36^ This was implemented in Python (version 3.12.7) using the open-source library scikit-learn (version 1.5.1).^37^ Afterward, the preprocessed data was imported in SIMCA (Sartorius AG, Germany) to construct a orthogonal partial least-squares-discriminant analyses (OPLS-DA) model according to previously described details.^26^ Furthermore, support vector machine (SVM), random forest classifier (RF), and multilayer perceptron (MLP) models were implemented using the Python library scikit-learn (version 1.5.1).^37^ The hyperparameters of the machine learning models were selected with Bayesian optimization as implemented in the Python library scikit-optimize (version 0.10.2). We let the algorithm explore 100 parameter combinations and selected the best one through 5-fold cross-validation. A list of the selected hyperparameters for each benchmark model can be found in Supporting Table 4.

We also compared LCMS-Net against DeepMSProfiler, a state-of-the art deep learning model for the analysis of raw LC-HRMS data.^14^ Similarly to LCMS-Net, the first step of DeepMSProfiler is the binning of the raw LC-HRMS data. However, DeepMSProfiler uses a fixed binning length and repeats the data across three input channels. Afterward, a pretrained DenseNet-121 architecture with 2D convolutions is used for the classification task. Thus, DeepMSProfiler performs smoothing over both the RT- and *m*/*z*-axis and initially relies on features that were extracted from natural images. DeepMSProfiler was implemented based on the publicly available source code from Deng et al.^14^ The data handling pipeline was updated for computational efficiency due to the comparatively large size of the CoD screening dataset used in this study. However, the model architecture itself was left unchanged. The same Bayesian optimization setup as for LCMS-Net was used to select the learning rate and optimizer settings for the training of DeepMSProfiler. A list of the selected hyperparameters can be found in Supporting Table 5.

#### Evaluation Metrics

To assess the prediction performance of LCMS-Net and the baseline models, we used accuracy, sensitivity, specificity, and F1-score. These classification metrics can be derived from the number of true positives (TP), true negatives (TN), false positives (FP), and false negatives (FN) produced by a classifier. In a binary classification problem, TPs correspond to positive instances that are correctly identified as such (i.e., *y* = *ŷ* and *y* = 1, where *y* denotes the true class label and *ŷ* the predicted class label). Similarly, TNs are negative instances correctly classified as negative (i.e., *y* = *ŷ* and *y* = 0), FPs are negative instances falsely classified as positive, and FNs are positive instances falsely classified as negative. As accuracy reflects the proportion of all correct predictions of a classifier, it can be formulated as

$$A\mathrm{ccuracy}=\frac{\mathrm{TP}+\mathrm{TN}}{\mathrm{TP}+\mathrm{TN}+\mathrm{FP}+\mathrm{FN}}$$ 1

Sensitivity (also known as recall) measures how effectively a model detects positive instances. It is defined as the fraction of true positives among all positive instances

$$\mathrm{Sensitivity}=\frac{\mathrm{TP}}{\mathrm{TP}+\mathrm{FN}}$$ 2

Specificity, in contrast, measures a classifier’s ability to correctly identify negative instances. It is defined as

3 $$\mathrm{Specificity}=\frac{\mathrm{TN}}{\mathrm{TN}+\mathrm{FP}}$$

Lastly, the F1-score is more suitable as a metrics for data with unbalanced class distribution and reflects the balance between the classifier’s ability to correctly identify positive instances and to minimize misclassification. It is defined as

4 $$F1‐\mathrm{score}=\frac{2\mathrm{TP}}{2\mathrm{TP}+\mathrm{FP}+\mathrm{FN}}$$

Macro-averaging was used when reporting a classification metric over all output classes. For class-wise evaluations, a binary one-vs-rest approach was used. Furthermore, repeated trials with random initialization were used to account for variance between model runs. Small differences between iterations can be expected due to variations in the validation split used for early stopping, data augmentation techniques, and random weight initialization. However, large differences may suggest a lack of robustness. All evaluation metrics were computed using Python (version 3.12.7) with the open-source libraries scikit-learn and imbalanced-learn^37,38^

#### Model Comparison

Furthermore, Wilcoxon signed-rank test was used to test whether a baseline model outperforms LCMS-Net with respect to the chosen evaluation metrics. This approach was previously suggested for statistical comparison of results of machine learning models.^39,40^ The nonparametric test ranks the performance difference *d* of two classifiers based on *N* datasets and compares the ranks of positive and negative differences. The tests statistic can be formulated as

5 $$T=\min\left\{R^{+},R^{-}\right\},\;\mathrm{where}\;R^{+}=\sum_{d_{i}>0}\mathrm{rank}\left(|d_{i}|\right),\;R^{-}=\sum_{d_{i}<0}\mathrm{rank}\left(|d_{i}|\right)$$

where *d*_*i*_ refers to the difference of two classifiers on the *i*-th dataset.^39,40^ The *T*-statistic can be examined by comparing it to its critical values (either exact or normal estimation).

To perform Wilcoxon signed-rank test, stratified 10-fold cross-validation was used to create the necessary datasets for the model comparison. Afterward, each model is trained with 9 cross-validation folds and evaluated on the remaining cross-validation fold. The SciPy (version 1.13.1) implementation of Wilcoxon signed-rank test was used to compute the test statistics and p-values in Python.^41^ Furthermore, the Benjamini–Hochberg procedure was used to compensate for multiple comparisons.^39^

## Results and Discussion

### Evaluation of Prediction Performance for CoD Screening

First, LCMS-Net was evaluated on the CoD screening dataset (Dataset A). Figure 2 shows the results of the model comparison for the CoD screening dataset. With an average accuracy of 70.8%, LCMS-Net outperformed all four benchmark models trained on preprocessed LC-HRMS data. In comparison, our previously published OPLS-DA model for CoD screening achieved only 65.0% accuracy (*p*-value = 0.0010). Furthermore, LCMS-Net also achieved a better F1-score and sensitivity than these models, while maintaining a high specificity score. The deep learning benchmark model, DeepMSProfiler, achieved a similar accuracy to LCMS-Net. However, LCMS-Net outperformed DeepMSProfiler in terms of both F1-score (*p*-value = 0.0122) and sensitivity (*p*-value = 0.0010). This indicates that our method maintains a better balance between true positives and false positives across the five CoD groups, which is especially important for high-stakes applications like forensic science. It should be noted that traditional cross-validation was used for the estimation of p-values, as our purpose was to compare models with fixed hyperparameters. When instead comparing frameworks (e.g., RF vs SVM), nested cross-validation would be more appropriate.

**Figure 2:**
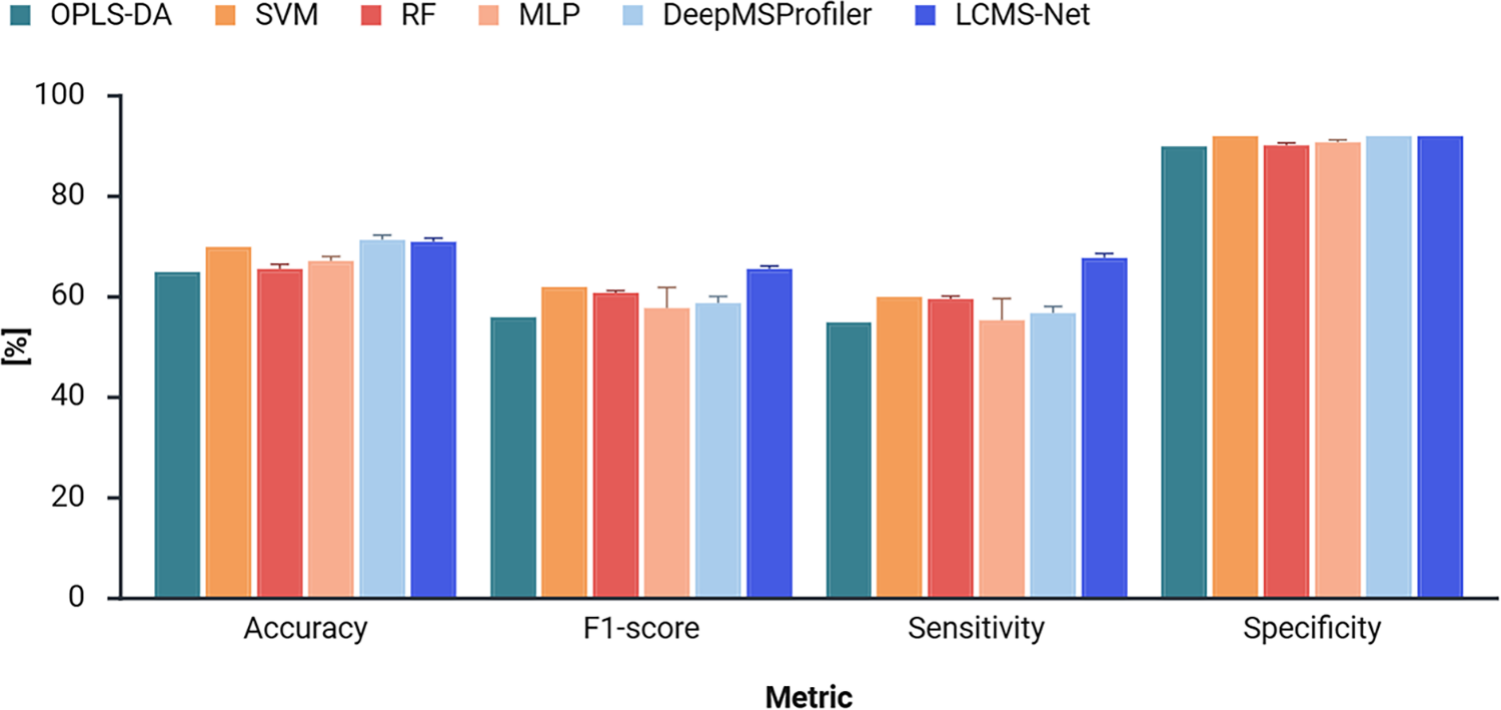
Evaluation of CoD screening prediction performance of LCMS-Net in comparison to benchmark models. The prediction performance of a classifier is measured by accuracy, macro F1-score, macro sensitivity, and macro specificity over five model runs. The error bars represent the standard deviations between model runs.

Notably, LCMS-Net achieves its high classification performance without pretraining on natural images. This shows that although transfer learning is often useful, it may fail if the source (e.g., natural images) and target data distribution (e.g., LC-HRMS data) are too different. Such domain mismatch can lead to negative transfer, where features from a pretrained model negatively impact the classification performance of the downstream task, resulting in a lower accuracy than training a simpler model from scratch.^42^ By employing an architecture tailored to LC-HRMS data, LCMS-Net is also computationally more efficient, with fewer than 500,000 trainable parameters compared to over 7 million trainable parameters in DeepMSProfiler.^14^ This makes our method accessible to researchers with limited computational resources.

For a more detailed evaluation of the results, we also compared the prediction performance of LCMS-Net and the benchmark models across the individual CoD groups. For conciseness we focused the class-wise comparison only on OPLS-DA and SVM. OPLS-DA was selected as it is the current state-of-the-art model for CoD screening, while SVM was included due to its good overall performance. All three models exhibit noticeable performance differences across the five CoD groups. Drug intoxication and hanging cases were overall well-classified, whereas IHD and pneumonia presented the greatest challenges. This was particularly evident in the F1-scores and sensitivity values. For pneumonia cases, OPLS-DA and SVM achieved F1-scores below 30% and sensitivity scores below 20%, indicating a limited ability to correctly predict the CoD groups. Although LCMS-Net also struggled to predict pneumonia cases, it showed considerable improvements compared to the benchmark models, with an F1-score of 34.9% and a sensitivity of 46.1%. Similarly, large improvements were observed for acidosis cases, where LCMS-Net achieved an F1-score of 71.7% and a sensitivity of 76.0%. Furthermore, the CoD group-specific accuracy scores are notably higher than the overall accuracy. This is due to the use of a binary one-vs-rest approach for computing the class-wise evaluation metrics, in which one CoD group (“positive” group) is discriminated against all remaining CoD groups (“negative” group). This introduces a substantial class imbalance, which can result in high accuracy scores by correctly classifying the“negative” group rather than the target CoD group. In contrast, the overall accuracy is computed by discriminating among all five CoD groups. An overview of the class-wise comparison is shown in Figure 3.

**Figure 3:**
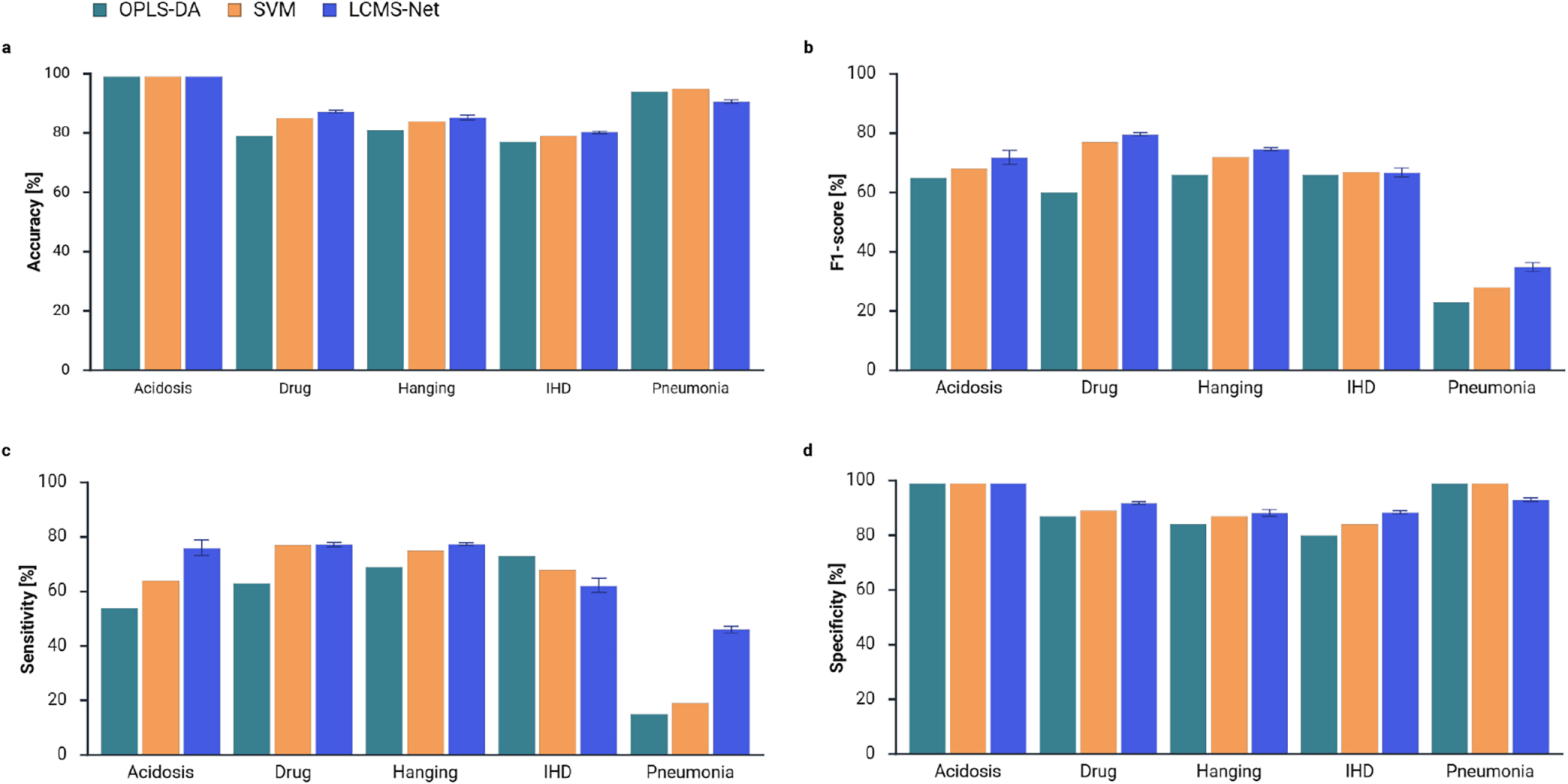
Class-wise evaluation of the prediction performance of LCMS-Net in comparison to OPLS-DA and SVM. The evaluation metrics are computed by treating one class as the“positive class” and all others as“negative”. Thus, the multiclass classification task was reformulated as a binary problem. The following metrics were computed for the five CoD groups (a) accuracy, (b) macro F1-score, (c) macro sensitivity, and (d) macro specificity. Error bars are used to indicate the standard deviation over five model runs.

As acidosis and pneumonia are the most underrepresented CoD groups in our dataset, these results also indicated that the end-to-end deep learning model effectively captures informative patterns and generalizes well, even when training data is limited, which is a frequent constraint of machine learning applications in forensic medicine. Lastly, it is worth to mention that IHD is the only CoD group for which some of the tested benchmark models achieved slightly better F1-scores and sensitivity than LCMS-Net. However, this comes at the cost of lower specificity.

### False-Positives Investigation

In order to assess potential limitations of LCMS-Net, we also performed an analysis of misclassified cases of the CoD screening dataset. Although false positives occurred across all five CoD groups, the majority of misclassified cases were either predicted as drug intoxication or IHD. This may be connected to contributing CoD diagnoses, that overlap with the five studied CoD groups. Generally, a contributing CoD is defined as a condition present at the time of death that is connected to the outcome but not directly part of the chain of morbid events. In the pneumonia group, the influence of contributing CoDs on false positive predictions is particularly evident. About 25% of all samples falsely classified as drug intoxication have drug intoxication listed as a contributing CoD. A similar trend can also be observed for IHD, which is overall the most frequent contributing CoD out of the five defined groups. Lastly, our analysis reveals that 69% of all pneumonia cases and 56% of all acidosis cases in the CoD screening dataset were diagnosed with at least one contributing CoD. This indicates substantial intraclass variability, which may affect the classification performance of LCMS-Net by obscuring class boundaries, especially in the presence of low sample sizes. Figure 4 summarizes the results of the false-positive investigation, by depicting the predicted class labels for each CoD group, along with the associated contributing CoD diagnoses.

**Figure 4:**
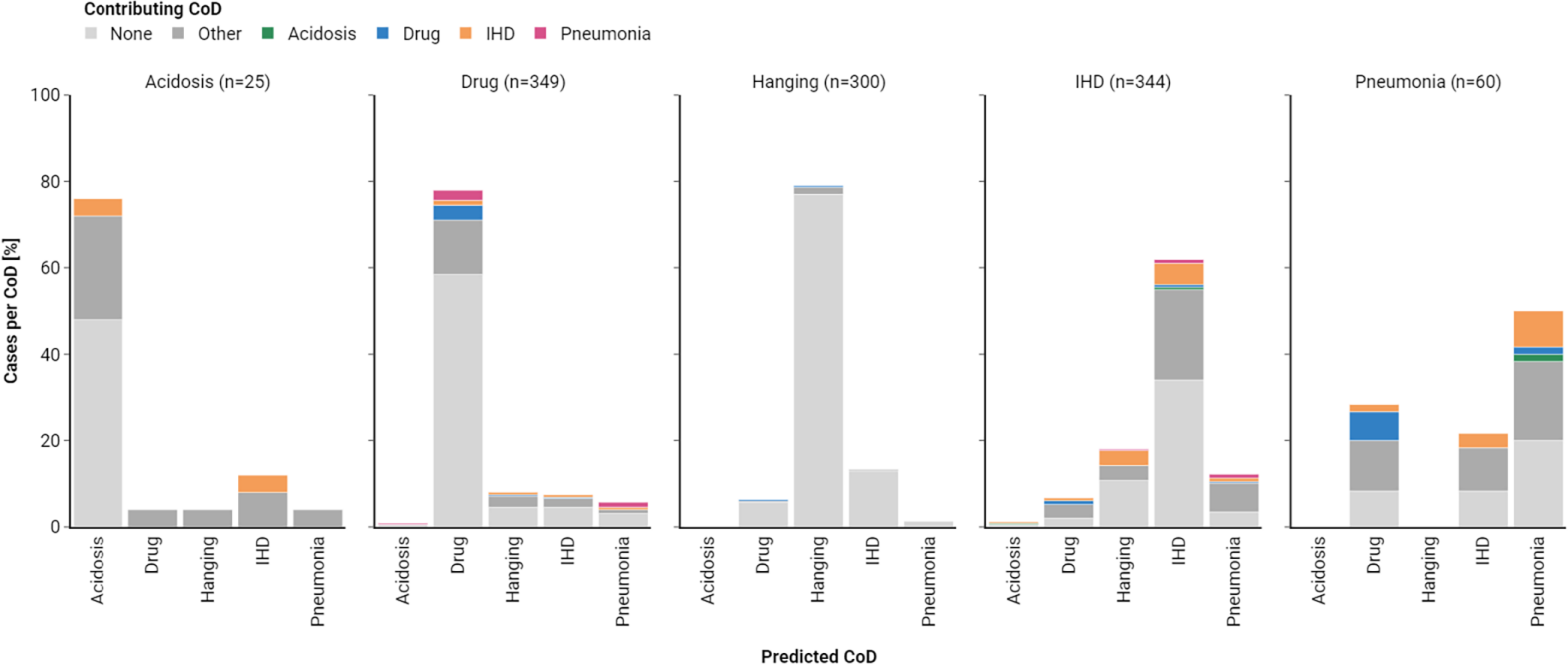
Analysis of false-positive predictions by contributing CoD diagnosis. For each CoD group, the proportions of predictions within that group are summarized. Colors represent the contributing CoD diagnoses and include the five studied CoD groups, as well as the additional categories “other CoD” (dark gray) and “no contributing CoD” (light gray). Since hanging can not be considered a contributing CoD, it was excluded from the color scale.

### Specificity Optimization

To reduce the number of false-positives, we performed specificity optimization. By default, LCMS-Net assigns a sample to the class with the highest predicted probability score. However, this approach does not consider the model’s confidence in its predictions, which is crucial for decision-making in high-stakes applications such as CoD screening.^43^ To address this and reduce the risk of misclassification, a reject option was implemented. Specifically, a sample is rejected if the model’s confidence, which is measured by the difference between the top two predicted class probabilities, is below a set threshold. We used 10% of the training data as a validation set to select the optimal threshold with respect to the specificity. After the selected 20% threshold was applied on the test set, LCMS-Net’s specificity improved by 4% compared to using the default class label assignment. However, this comes at the cost of lower accuracy, F1-, and sensitivity scores as approximately 27% of all cases were rejected and thus not assigned a CoD group. The majority of rejected cases were diagnosed as IHD or pneumonia, which is consistent with our prior findings that these CoD groups are particularly difficult to predict. A detailed overview of the results of the specificity optimization can be found in Supporting Table 6.

### Robustness toward Batch Effects

We also tested LCMS-Net’s robustness toward batch effects on the CoD screening dataset. For this purpose we performed two experiments with Dataset A and B: (1) We trained LCMS-Net with 75% of Dataset B and tested the predictive performance with the remaining 25% of cases. Afterward, we compared the results with those of Dataset A to evaluate if LCMS-Net performs differently on the two data batches when given the same task. (2) We trained LCMS-Net with Dataset A and tested the predictive performance on Dataset B and vice versa. A transferable model is expected to achieve comparable results on all these tasks despite the different sample collection periods and measurement instruments.

UMAP clustering of the binned LC-HRMS data (i.e., LCMS-Net’s input) shows that Dataset A and B can be clearly separated from each other, which indicates strong batch effects before the analysis with LCMS-Net (see Supporting Figure 2a). However, when tested on both datasets LCMS-Net maintains a comparable accuracy for both subsets of the CoD screening data. Furthermore, LCMS-Net exhibits only a minor drop in predictive performance when trained and tested with samples from the different datasets. We hypothesize that this is due to the use of data augmentation techniques, which enforce a robust representation of learned features. This robustness of results and transferability between measurement instruments helps to replicate findings across studies and potentially opens up new opportunities for collaborations between research groups or institutes. Figure 5 summarizes the results of the robustness test of LCMS-Net. Furthermore, Supporting Figure 2b shows the clustering of Dataset A and B based on feature representations extracted from the convolutional block of LCMS-Net (i.e., the last layer before classification layer).

**Figure 5:**
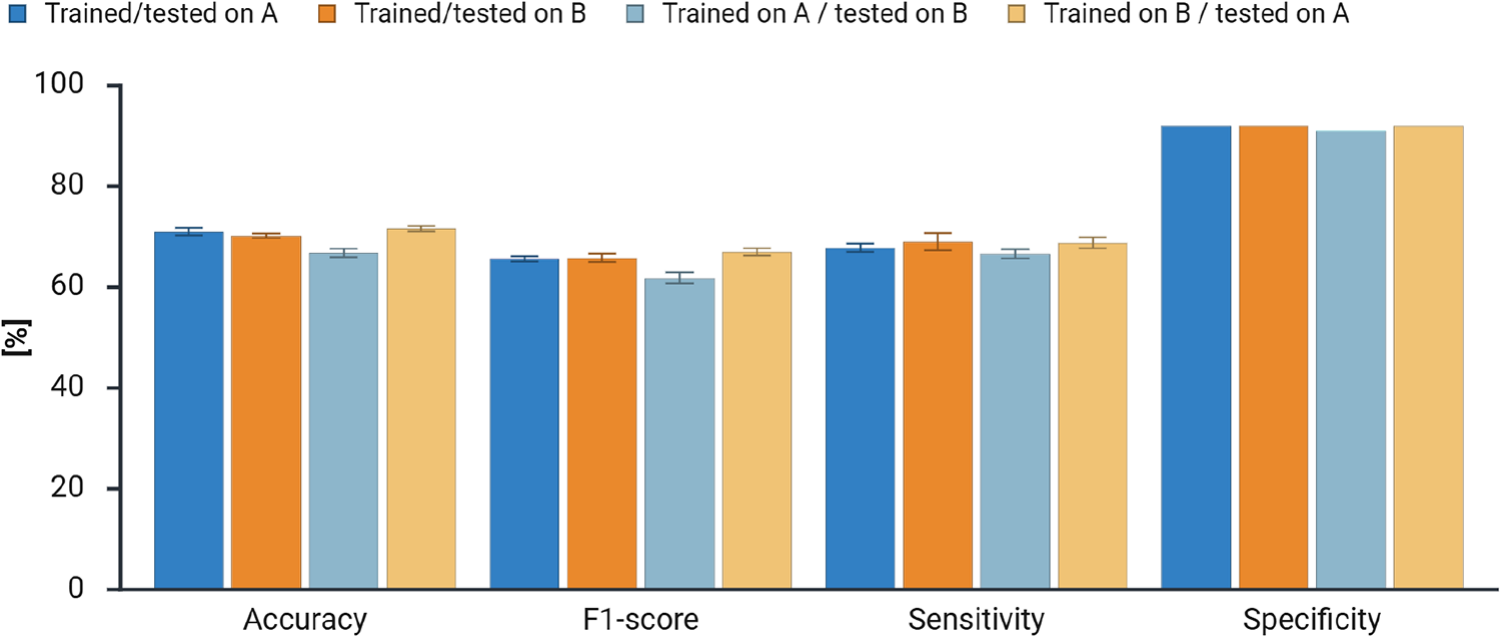
Evaluation of the impact of batch effects on the prediction performance of LCMS-Net. As before, the predictive performance is measured by accuracy, F1-score, sensitivity, and specificity over five model runs, and error bars indicate the standard deviation.

### Application for Colon Cancer Detection

Lastly, we have tested the potential of LCMS-Net in another application domain, by assessing its prediction performance on the CRC dataset. The cancer detection task has been previously used by Deng et al. to show that DeepMSProfiler can be successfully applied to different application areas, making it a suitable benchmark task. We trained a separate instance of LCMS-Net for the model evaluation on the CRC dataset due to differences in sample preparation, analytical protocols, and instrument setup compared to the CoD screening dataset. The hyperparameter settings remained unchanged, as we assumed similar structural properties for both datasets.

LCMS-Net outperforms DeepMSProfiler with an F1-score of 97.3% compared to 95.5% on the colon cancer detection task. Furthermore, sensitivity and specificity improvements of 2.8% were observed. These results underline the relevance of LCMS-Net for other application areas than CoD screening. A detailed overview of the evaluation metrics for the colon cancer prediction can be found in Supporting Table 7.

### Explainability of Results

A limitation of LCMS-Net is the explainability of results. Currently, it is not possible to investigate which metabolites are significant for a prediction, which hinders biological interpretations of the end-to-end deep learning model. In the future, this could be addressed through perturbation-based methods like randomized input sampling for explanation of black-box models (RISE) as suggested by Deng et al.^14^ RISE computes feature contribution by randomly probing a model with masked versions of the input data and analyzing the corresponding outputs to retrieve relevant features.^44^

## Conclusion

Current preprocessing workflows for untargeted metabolomics are often time-consuming, require extensive domain knowledge, lack reproducibility, or fail to detect some metabolites entirely. LCMS-Net offers an alternative end-to-end approach by operating directly on the raw LC-HRMS data and explicitly modeling its spatial properties. The deep learning model enables faster processing and does not require manual parameter selection. Furthermore, it has been shown through two case studies, CoD screening and cancer detection, that our method extracts a higher amount of relevant information from the raw data and thus outperforms existing models for the analysis of metabolomics data. Specifically, LCMS-Net achieves an average F1-score of 65.5% for CoD screening and 97.3% for colon cancer detection. The previous state-of-the-art models achieved 56.1% (OPLS-DA) and 95.5% (DeepMSProfiler) respectively. The prediction performance of LCMS-Net is consistent even when applied to data from different measurement instruments than those used for the training of the deep learning model, showing its robustness toward batch effects. However, the interpretability of LCMS-Net remains limited and future development is needed to assess which metabolites are learned by the model and which remain undetected. Despite this, LCMS-Net opens up new opportunities for large-scale analyses of LC-HRMS data, potentially driving innovation across numerous application domains.

## Supplementary Material



## Data Availability

The raw mass spectrometry data for CoD screening reported in this study cannot be deposited in a public repository because of ethical restrictions on the reporting of data derived from routine investigation of deceased individuals. Preprocessed metabolomics data and summary data reported in this paper can be shared by the lead contact upon reasonable request. The R code used to generate a feature list from the raw LC-HRMS data for the benchmark models is available in the Supporting Information (see Supporting Code 1) and was previously published open-access.^21^ Furthermore, all source code for LCMS-Net was made available on GitHub (https://github.com/lisamenacher/LCMS-Net) for academic use.

## Notes

Binning of raw LC-HRMS data (Text S1); design and implementation of LCMS-Net (Text S2); feature extraction with XCMS (Code S1); impact of the number of ensemble members on the validation prediction performance of LCMS-Net (Figure S1); UMAP clustering of datasets A and B (Figure S2); effect of different (adaptive) binning sizes on the prediction performance of LCMS-Net for the validation set (Table S1); summary of the ablation study for the model design of LCMS-Net (Table S2); hyperparameters for LCMS-Net (Table S3); hyperparameters for benchmark models on preprocessed data (Table S4); hyperparameters for DeepMSProfiler (Table S5); evaluation of LCMS-Net’s prediction performance after the specificity optimization (Table S6); evaluation of the performance of LCMS-Net for the colon cancer dataset in comparison to DeepMSProfiler (Table S7) (PDF)
