# Supplementary material for: LCMS-Net: Deep Learning for Raw High Resolution Mass Spectrometry Data Applied to Forensic Cause-of-Death Screening
Source: Anal Chem. 2026 Feb 27;98(9):6589–97. doi: 10.1021/acs.analchem.5c05404 (PMC12980486; doi:10.1021/acs.analchem.5c05404)
Supplement: Supplementary file 1 [file ac5c05404_si_001.pdf]

Supporting Information

for

LCMS-Net: Deep learning for raw high resolution mass spectrometry data applied to forensic cause-of-death screening

Lisa M. Menacher[1], Liam J. Ward[2,3], Fredrik Heintz[1,4], Henrik Green[2,3,5], and Oleg Sysoev[1]

1 Department of Computer and Information Science, Linköping University, 581 83 Linköping, Sweden
2 Department of Biomedical and Clinical Sciences, Linköping University, 581 83 Linköping, Sweden
3 Department of Forensic Genetics and Forensic Toxicology, National Board of Forensic Medicine, 587 58 Linköping, Sweden
4 AI4x Center of Excellence, Linköping University, 581 83 Linköping, Sweden
5 Department of Biomedical and Clinical Sciences, Science for Life Laboratory, Linköping University, 581 83 Linköping, Sweden

Table of Contents

Supplementary Text 1: Binning of raw LC-HRMS data S3

Supplementary Text 2: Design and implementation of LCMS-Net S3

Supplementary Code 1: Feature extraction with XCMS S5

Supplementary Figure 1: Impact of the number of ensemble members on the validation prediction performance S6

Supplementary Figure 2: UMAP clustering of datasets A and B S6

Supplementary Table 1: Effect of different (adaptive) binning sizes on the prediction performance of LCMS-Net for the validation set S7

Supplementary Table 2: Summary of ablation study for the model design of LCMS-Net S7

Supplementary Table 3: Hyperparameters for LCMS-Net S8

Supplementary Table 4: Hyperparameters for benchmark models on preprocessed data S8

Supplementary Table 5: Hyperparameters for DeepMSProfiler S9

Supplementary Table 6: Evaluation of LCMS-Net's prediction performance after the specificity optimization S9

Supplementary Table 7: Evaluation of the performance of LCMS-Net for colon cancer dataset in comparison to DeepMSProfiler S9

Supplementary Text 1 | Binning of raw LC-HRMS data

For the end-to-end deep learning, data binning was applied to the raw LC-HRMS data to reduce its size and provide a consistent input structure. During this process, data points within the injection phase (i.e., points with RT ≤ 45 seconds for all used datasets in this study) and wash-out phase (i.e., points with RT ≥ 660 seconds for all used datasets in this study) are excluded to avoid low-quality signals. If traditional/fixed binning is used, the remaining data points are mapped onto a predefined grid, which is constructed by partitioning the RT- and m/z-axis into n bins of equal width. Afterwards, all data points within a bin are aggregated by selecting the maximum intensity. The shape of the resulting data matrix is $n \times n$. Alternatively, if adaptive binning is used, prior knowledge about the LC-HRMS data is utilized to define the resolution (i.e., width) of each bin individually. The width of each m/z-bin is defined so that regions with a high expected number of metabolites are divided into finer-grained bins, while regions with fewer expected metabolites are split into broader bins. Specifically, the m/z-axis is divided into intervals of 50 Da, and the number of metabolites (retrieved from previous experiments on the same method) is counted within each interval. The fraction of metabolites falling into a given 50 Da interval is then used to determine how many bins (out of $n_{m/z}$ bins) are allocated to that interval. The total number of m/z-bins $n_{m/z}$ is a user-defined parameter. Supplementary Table 1 studies the influence of the number of m/z-bins on the prediction performance of LCMS-Net. The total number of RT-bins n_{RT} is also a user-defined parameter. However, in practice it can be set to match the sampling interval of the used measurement instrument (after removing noisy periods). This ensures that each bin corresponds to exactly one scan. The resulting binning grid of shape $n_{RT} \times n_{m/z}$ is then used to aggregate the data points within each bin by selecting the maximum intensity value.

The number of bins n for the fixed binning was set to 1,024 based on results from previous studies. Thus, if fixed binning is used (e.g., for DeepMSProfiler) the input matrices are of shape $1{,}024 \times 1{,}024$. The number of m/z-bins $n_{m/z}$ for adaptive binning was also set to 1,024 for all results presented in the main text. This number showed the best results on the validation set compared to smaller or larger binning grids (see Supplementary Table 1). The number of RT-bins n_{RT} for adaptive binning was set to 856 for both CoD screening datasets, as this is the number of scans obtained from the Agilent 6550 iFunnel LC/Q-TOF system (Dataset A) after excluding noisy periods. The Agilent 6546 Q-TOF system used for Dataset B produces more scans, but was down-scaled for the robustness study to match the input format of Dataset A. For the colon cancer dataset, the number of RT-bins n_{RT} was set to 1,954 for the adapting binning to match the number of scans produced by the respective measurement instrument after removing noisy periods. Thus, if adaptive binning is used (e.g., for LCMS-Net) the input matrices are of shape $856 \times 1{,}024$ for the CoD screening datasets and of shape $1{,}954 \times 1{,}024$ for the colon cancer dataset.

Supplementary Text 2 | Design and implementation of LCMS-Net

LCMS-Net uses depthwise 1D convolutions, 1D pooling, and spatial dropout to capture the spatial properties of LC-HRMS data. We verified these architectural choices on the CoD screening dataset through an ablation study using a simple base architecture consisting of one convolutional, pooling, dropout, and dense layer. Furthermore, we also tested the impact of the

binning method, different class balancing approaches, normalization techniques, and data augmentation. The contribution of each component was evaluated over five model runs using 10% of the training data as a validation set. Stratified sampling was again used to maintain class proportions. Supplementary Table 2 provides an overview of the results of the ablation study. Based on these results, we selected depthwise 1D convolutions along the RT-axis, max pooling, adaptive binning, and min-max normalization along the RT-axis for LCMS-Net. Furthermore, random oversampling (ROS) and data augmentation are used during the training of the deep learning model.

Next, Bayesian optimization was used to select the hyperparameters of LCMS-Net. This includes the number of convolution blocks and dense layers, the kernel size and stride length of each convolutional layer, the size and stride length of each pooling layer, the rate of each dropout layer, the number of hidden units and regularization of each dense layer, and the optimizer settings. KerasTuner with default priors was used to implement the optimization workflow.[1] The maximum number of trials was set to 100, allowing a trade-off between the exploration of different hyperparameter configurations and computational efforts. A list of the selected hyperparameters can be found in Supplementary Table 3. All weights of LCMS-Net were initialized with the He-Normal function.[1] Furthermore, early stopping with a patience of 10 epochs was used to prevent overfitting, and a learning rate scheduler was used to reduce the learning rate by a factor of 0.5 when the validation loss did not improve for three consecutive epochs.[1]

Lastly, an ensemble strategy was adopted, as the observed variation of the prediction performance on the validation set between model runs was relatively large. Thus, multiple instances of LCMS-Net are trained, and their predicted class probabilities are averaged to obtain the final predictions. Class labels are then assigned by selecting the class with the highest average predicted probability. In this study, we used 11 ensemble members for all experiments, as this provided the best prediction performance on the validation set (see Supplementary Fig. 1).

[1] Chollet, F. et al. Keras https://keras.io. 2015.524

Supplementary Code 1 | Feature extraction with XCMS

```
library("xcms")
library("CAMERA")
xset <- xcmsSet(method="centWave",
     ppm=30,peakwidth=c(3,20),snthresh=3, noise=1000)
xset<-group(xset, mzwid=0.05,bw=3)
xset2<-retcor(xset,method="obiwarp",response=10,plottype="d")
xset2<-group(xset2, mzwid=0.05,bw=3,minfrac=0.5)
xset3<-fillPeaks(xset2)
an<-xsAnnotate(xset3)
anF<-groupFWHM(an, perfwhm=0.6)
anC<-groupCorr(anF)
anFI<-findIsotopes(anC)
anFA<-findAdducts(anFI, polarity="positive")
write.csv(getPeaklist(anFA), file = "result_table.csv")
```

Supplementary Figure 1 | Impact of the number of ensemble members on the validation prediction performance.

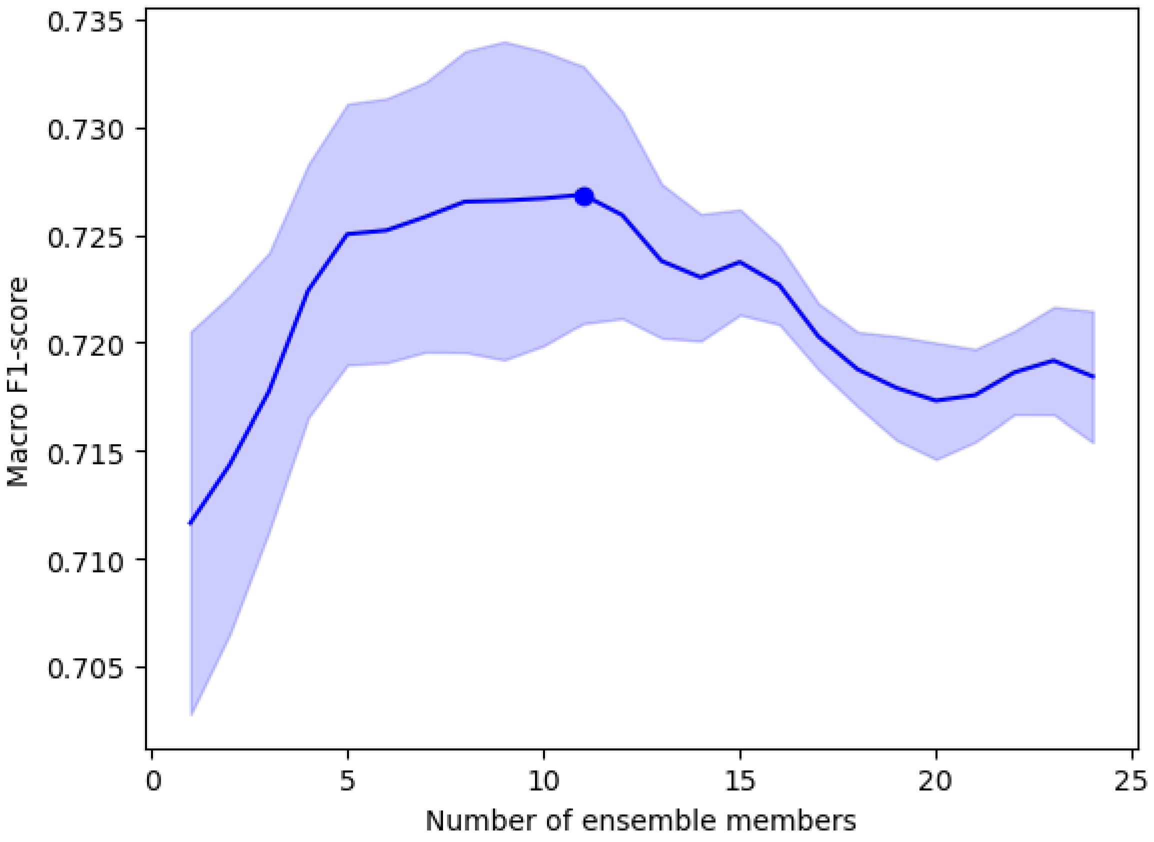

Supplementary Figure 2 | UMAP clustering of datasets A and B. (a) Clustering based on binned LC-HRMS samples (i.e., input of LCMS-Net). (b) Clustering based on the feature representations extracted from the convolutional block of LCMS-Net (i.e., the last layer before the classification layer).

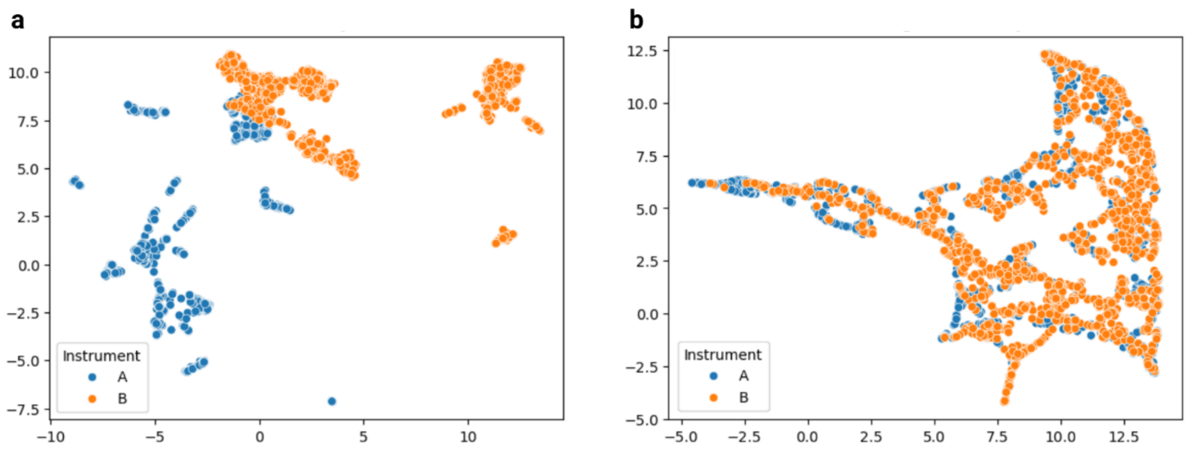

Supplementary Table 1 | Effect of different (adaptive) binning sizes on the prediction performance of LCMS-Net for the validation set.

Binning size	Accuracy	F1-score	Sensitivity	Specificity
214 × 256	62.9 ± 3	56.2 ± 2	55.7 ± 4	89.6 ± 1
428 × 512	64.0 ± 2	57.9 ± 2	57.6 ± 2	89.9 ± 1
856 × 1024	66.6 ± 1	63.1 ± 1	63.2 ± 2	90.7 ± 0
856 × 2375	66.9 ± 10	62.5 ± 10	65.5 ± 11	91.0 ± 3
856 × 4750	65.9 ± 8	61.6 ± 8	61.7 ± 10	90.6 ± 2

Supplementary Table 2 | Summary of ablation study for the model design of LCMS-Net.

		Accuracy	F1-score	Sensitivity	Specificity
Convolution	1D (RT-axis)	64.9 ± 2	60.9 ± 2	60.4 ± 2	90.1 ± 0
	1D (m/z-axis)	62.8 ± 2	55.9 ± 3	56.1 ± 5	89.6 ± 0
	2D	63.9 ± 2	58.9 ± 2	58.7 ± 3	89.8 ± 0
Pooling	Avg.	66.6 ± 3	62.4 ± 5	61.9 ± 7	90.6 ± 1
	Max.	64.9 ± 2	60.9 ± 2	60.4 ± 2	90.1 ± 0
Binning	Fixed	64.9 ± 2	60.9 ± 2	60.4 ± 2	90.11 ± 0
	Adaptive	66.6 ± 1	63.1 ± 1	63.2 ± 2	90.7 ± 0
Class balancing	None	64.9 ± 2	60.9 ± 2	60.4 ± 2	90.1 ± 0
	Weighting	64.5 ± 3	60.4 ± 4	62.4 ± 5	90.1 ± 1
	ROS	65.8 ± 3	61.8 ± 3	64.3 ± 4	90.5 ± 1
Augmentation	None	64.9 ± 2	60.9 ± 2	60.4 ± 2	90.1 ± 0
	Yes	66.3 ± 4	66.8 ± 3	66.5 ± 3	90.5 ± 1
Normalization	None	64.9 ± 2	60.9 ± 2	60.4 ± 2	90.1 ± 0
	Sample-wise	48.9 ± 3	30.7 ± 2	32.0 ± 2	85.22 ± 1
	Along RT-axis	67.6 ± 1	53.4 ± 6	50.9 ± 4	90.7 ± 0
	Along m/z-axis	73.1 ± 2	68.2 ± 2	66.0 ± 3	92.3 ± 1

Supplementary Table 3 | Hyperparameters for LCMS-Net.

	Hyperparameter	Value
Other	Ensemble members	11
	Class balancing	Random oversampling
	Augmentation	True
Optimizer	Batch size	4
	Learning rate	0.0001
	Weight decay	0.11
	Amsgrad	False
	Epsilon	0.005
	Beta 1	0.09
	Beta 2	0.999
	Number of conv.-blocks	1
Convolution	Kernel size	42
	Kernel stride	5
	Kernel regularization	L1: 0.01, L2: 0.05
Pooling	Pooling size	6
	Pooling stride	2
Spatial dropout	Rate	0.1
Dense layers	Number of dense-layers	1
	Regularization	L1: 0.002, L2: 0.1

Supplementary Table 4 | Hyperparameters for benchmark models on preprocessed data.

	Hyperparameter	Value
Random forest	Criterion	entropy
	Bootstrap	False
	Class weight	balanced
	Max. depth	85
	Max. features	/
	Max. leaf nodes	200
	Min. samples leave	2
	N estimators	250
Support vector machine	C	1083
	Degree	8
	Gamma	1e-06
Multi-layer perceptron	Hidden units (layer 1)	79
	Hidden units (layer 2)	351
	Hidden units (layer 3)	48
	Batch size	128
	Early stopping	True
	Learning rate	0.006 (with invscaling)
	Epsilon	0.05
	Alpha	2.83
	Beta 1	0.82
	Beta 2	0.84

Supplementary Table 5 | Hyperparameters for DeepMSProfiler.

Hyperparameter	Value
Ensemble members	18
Batch size	8
Learning rate	0.0005
Weight decay	0.81
Amsgrad	False
Epsilon	0.15
Beta 1	0.75
Beta 2	0.94

Supplementary Table 6 | Evaluation of LCMS-Net's prediction performance after the specificity optimization.

	Accuracy	F1-score	Sensitivity	Specificity
Acidosis	98.7	71.1	68.0	99.4
Drug intox.	86.5	76.8	69.1	94.8
Hanging	86.5	73.0	65.1	94.9
IHD	78.3	55.6	42.8	94.9
Pneumonia	92.2	35.5	35.9	95.4
Overall	57.7	62.0	56.2	95.9

Supplementary Table 7 | Evaluation of the performance of LCMS-Net for colon cancer dataset in comparison to DeepMSProfiler.

	Accuracy	F1-score	Sensitivity	Specificity
DeepMSProfiler	97.8	95.5	92.9	92.9
LCMS-Net	98.7	97.3	95.7	95.7
